# ﻿Morphology and phylogeny of *Cytospora* (Cytosporaceae, Diaporthales) species associated with plant cankers in Tibet, China

**DOI:** 10.3897/mycokeys.104.113567

**Published:** 2024-04-16

**Authors:** Jiangrong Li, Jieting Li, Ning Jiang

**Affiliations:** 1 Key Laboratory of Forest Ecology in Tibet Plateau, Ministry of Education,Institute of Tibet Plateau Ecology, Tibet Agricultual & Animal Husbandry University, Nyingchi, Tibet 860000, China Tibet Agricultual & Animal Husbandry University Nyingchi China; 2 National Key Station of Field Scientific Observation & Experiment, Nyingchi, Tibet 860000, China National Key Station of Field Scientific Observation & Experiment Nyingchi China; 3 Key Laboratory of Biodiversity Conservation of National Forestry and Grassland Administration, Ecology and Nature Conservation Institute, Chinese Academy of Forestry, Beijing 100091, China Key Laboratory of Biodiversity Conservation of National Forestry and Grassland Administration, Ecology and Nature Conservation Institute, Chinese Academy of Forestry Beijing China

**Keywords:** Ascomycota, molecular phylogeny, novel taxa, Sordariomycetes, taxonomy

## Abstract

During our biodiversity investigations in Tibet, China, typical Cytospora canker symptoms were observed on branches of hosts *Myricariapaniculate*, *Prunuscerasifera* and *Sibiraeaangustata*. Samples were studied, based on morphological features coupled with multigene phylogenetic analyses of ITS, *act*, *rpb2*, *tef1* and *tub2* sequence data, which revealed two new species (*Cytosporamyricicola***sp. nov.** and *C.sibiraeicola***sp. nov.**) and a known species (*C.populina*). In addition, *Cytosporapopulina* is newly discovered on the host *Prunuscerasifera* and in Tibet.

## ﻿Introduction

The genus *Cytospora* (Cytosporaceae, Diaporthales, Sordariomycetes, Ascomycota) was proposed by Ehrenberg (1818) and *C.chrysosperma* was selected as the lectotype later (Donk 1964). *Cytospora* has priority over *Leucocytospora*, *Leucostoma*, *Valsa*, *Valsella* and *Valseutypella* based on the dual-nomenclature criterion (Adams et al. 2005; Rossman et al. 2015). Members of *Cytospora* are characterised by the single or labyrinthine, loculate stromata, filamentous conidiophores or asci and allantoid hyaline conidia or ascospores (Spielman 1985; Adams et al. 2005; Norphanphoun et al. 2017, 2018; Fan et al. 2020; Shang et al. 2020). Species identification in *Cytospora* was previously largely based on the host affiliation and morphological descriptions; however, molecular phylogeny combined with morphology and host affiliation have became the main approaches recently (Fan et al. 2020; Shang et al. 2020; Zhu et al. 2020). Currently, over 690 species epithets of *Cytospora* have been listed in Index Fungorum (http://www.indexfungorum.org/; 2023). However, most of these were regarded as synonyms and most descriptions were unable to identify them accurately (Adams et al. 2005; Fan et al. 2020; Pan et al. 2020).

*Cytospora* is distributed worldwide and often known to be associated with plant diseases (Monkai et al. 2021; Pan et al. 2021; Lin et al. 2022; Travadon et al. 2022). For example, *C.chrysosperma* is the main canker disease pathogen of polar and willow trees in China (Fan et al. 2014; Wang et al. 2015; Lin et al. 2023); *C.kuanchengensis* and additional five species are associated with Chinese chestnut cankers (Jiang et al. 2020); Fifteen *Cytospora* species were identified from destructive canker and dieback pathogens of woody hosts in the USA (Lawrence et al. 2018).

The Tibet Tibetan Autonomous Region is located on the Qinghai-Tibet Plateau, which is known as the third pole of the earth. During our biodiversity investigations in Tibet, typical fruiting bodies of *Cytospora* were discovered from *Myricariapaniculate* (Tamaricaceae), *Prunuscerasifera* (Rosaceae) and *Sibiraeaangustata* (Rosaceae). The aim of the present study was to identify *Cytospora* species from the three hosts based on morphological features and molecular phylogeny of combined sequence data.

## ﻿Materials and methods

### ﻿Sample collection, morphology and isolation

Our biodiversity investigations were conducted in Lhasa and Shigatse cities in Tibet Tibetan Autonomous Region, China during 2022 and 2023. Diseased branches of *Myricariapaniculate*, *Prunuscerasifera* and *Sibiraeaangustata* were observed and collected, packed in paper bags and returned to the laboratory for morphological study and fungal isolation.

Observation and description of *Cytospora* species was based on fruiting bodies naturally formed on the host barks. Ascostromata and conidiomata from tree barks were sectioned by hand using a double-edged blade and structures were observed under a dissecting microscope. At least 10 conidiomata/ascostromata, 10 asci and 50 conidia/ascospores were measured to calculate the mean size and standard deviation. Measurements are reported as maximum and minimum in parentheses and the range representing the mean plus and minus the standard deviation of the number of measurements is given in parentheses. Microscopy photographs were captured with a Nikon Eclipse 80i compound microscope, equipped with a Nikon digital sight DS-Ri2 high definition colour camera, using differential interference contrast illumination.

Isolates of *Cytospora* were obtained by removing the spore masses from the fruiting bodies on to clean PDA plates and incubating at 25 °C until spores germinated. Single germinated spores were then transferred to the new PDA plates and incubated at 25 °C in the dark. The cultures were deposited in the China Forestry Culture Collection Center (CFCC, http://cfcc.caf.ac.cn/) and the specimens in the Herbarium of the Chinese Academy of Forestry (CAF, http://museum.caf.ac.cn/).

### ﻿DNA extraction, PCR amplification and sequencing

Genomic DNA was extracted from fresh fungal mycelia following the method described by Doyle and Doyle (1990). Polymerase chain reactions (PCR) were conducted to amplify the internal transcribed spacer region rDNA (ITS), the partial actin (act) region, RNA polymerase II second largest subunit (*rpb2*), translation elongation factor 1-alpha (*tef1*) and the partial beta-tubulin (*tub2*) gene using primers and conditions listed in Table 1. The PCR products were assayed via electrophoresis in 2% agarose gels. DNA sequencing was performed using an ABI PRISM 3730XL DNA Analyser with a BigDye Terminator Kit v.3.1 (Invitrogen, Waltham, MA, USA) at the Shanghai Invitrogen Biological Technology Company Limited (Beijing, China).

**Table 1.** Primers and PCR protocols.

Gene Regions	Primers	PCR conditions	References
ITS	ITS5/ITS4	95 °C for 4 min, 35 cycles of 94 °C for 45 s, 48 °C for 1 min, and 72 °C for 2 min , 72 °C for 10 min	White et al. (1990)
*act*	ACT512F/ACT783R	95 °C for 4 min, 35 cycles of 94 °C for 45 s, 55 °C for 1 min, and 72 °C for 2 min , 72 °C for 10 min	Carbone and Kohn (1999)
*rpb2*	fRPB2-5f/fRPB2-7cR	95 °C for 5 min, 35 cycles of 95 °C for 1 min, 55 °C, 1.25 min, and 72 °C for 2 min , 72 °C for 10 min	Liu et al. (1999)
*tef1*	983F/2218R	94 °C for 3 min, 35 cycles of 94 °C for 30 s, 54 °C for 50 s, and 72 °C for 2 min, 72 °C for 10 min	Rehner (2001)
*tub2*	Bt2a/Bt2b	95 °C for 4 min, 35 cycles of 94 °C for 45 s, 54 °C for 1 min, and 72 °C for 2 min , 72 °C for 10 min	Glass and Donaldson (1995)

### ﻿Sequence alignment and phylogenetic analyses

The obtained sequences of ITS, *act*, *rpb2*, *tef1* and *tub2* were assembled using SeqMan software version 7.1.0 (DNASTAR Inc., WI) and subjected to BLASTn search against the GenBank nucleotide database at National Center for Biotechnology Information (NCBI) to identify closely-related sequences. Sequences data of related taxa were obtained from previous publications (Fan et al. 2020; Lin et al. 2023) and downloaded from the GenBank database (Table 2). The sequences were aligned using MAFFT v.7 online web server (http://mafft.cbrc.jp/alignment/server/index.html, Katoh et al. 2019) under default settings. The Maximum Likelihood (ML) phylogenic analysis was run in the CIPRES Science Gateway platform (Miller et al. 2010), using RAxMLHPC2 on the XSEDE (v. 8.2.10) tool under the GTR substitution model and 1000 non-parametric bootstrap replicates. Bayesian analysis was performed using MrBayes v. 3.2.6 on XSEDE at the CIPRES Science Gateway with four simultaneous Markov Chain runs for 1,000.000 generations. The resulting trees were visualised in FigTree v. 1.4.0 (Rambaut 2012).

## ﻿Results

### ﻿Phylogenetic analyses

**Figure 1.**
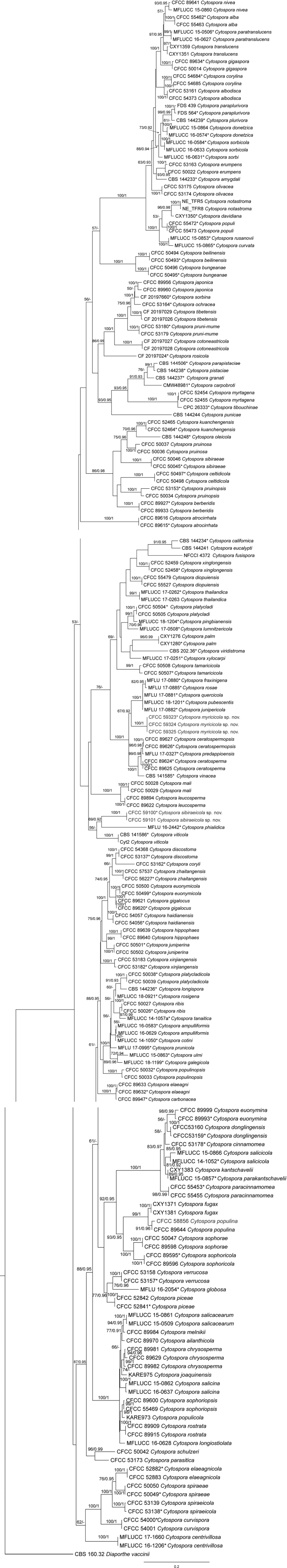
Maximum Likelihood tree generated from combined ITS, *act*, *rpb2*, *tef1* and *tub2* sequence data. Bootstrap support values ≥ 50% and Bayesian posterior probabilities ≥ 0.90 are demonstrated at the branches. Ex-type cultures are marked with (*).

The combined ITS, *act*, *rpb2*, *tef1* and *tub2* dataset consisted of 199 strains, with *Diaporthevaccinii* (CBS 160.32) as the outgroup taxon (Table 2). The final alignment comprised 3,166 characters (ITS: 567, *act*: 323, *rpb2*: 741, *tef1*: 727, *tub2*: 808), including gaps. The final ML optimisation likelihood value of the best RAxML tree was -60353.67 and the matrix had 2069 distinct alignment patterns, with 40.24% undetermined characters or gaps. Estimated base frequencies were as follows: A = 0. 244507, C = 0. 288246, G = 0. 237262, T = 0. 229984; substitution rates AC = 1.372283, AG = 2.995828, AT = 1.353835, CG = 0.976452, CT = 5.021434, GT = 1.0; gamma distribution shape parameter α = 0.372885. The RAxML and Bayesian analyses yielded a similar tree topology. The topology of our phylogenetic tree is nearly identical to previous publications. Six isolates from the present study formed two new clades distinct from previously-known species named *Cytosporamyricicola* sp. nov. and *C.sibiraeicola* sp. nov. and a known clade named *C.populina* (Fig. 1).

**Table 2.** GenBank accession numbers used in the phylogenetic analyses.

Species	Strain	Host	Origin	GenBank accession numbers				
				ITS	*act*	*rpb2*	*tef1*	*tub2*
*Cytosporaailanthicol*a	CFCC 89970	*Ailanthusaltissima*	China	MH933618	MH933526	MH933592	MH933494	MH933565
*Cytosporaalbodisca*	CFCC 53161	*Platycladusorientalis*	China	MW418406	MW422899	MW422909	MW422921	MW422933
*Cytosporaalbodisca*	CFCC 54373	*Platycladusorientalis*	China	MW418407	MW422900	MW422910	MW422922	MW422934
*Cytosporaalba*	CFCC 55462^T^	*Salixmatsudana*	China	NR182387	OK303457	OK303516	OK303577	OK303644
*Cytosporaalba*	CFCC 55463	*Salixmatsudana*	China	MZ702596	OK303458	OK303517	OK303578	OK303645
*Cytosporaampulliformis*	MFLUCC 16-0583^T^	*Sorbusintermedia*	Russia	KY417726	KY417692	KY417794	NA	NA
*Cytosporaampulliformis*	MFLUCC 16-0629	*Acerplatanoides*	Russia	KY417727	KY417693	KY417795	NA	NA
*Cytosporaamygdali*	CBS 144233^T^	*Prunusdulcis*	USA	MG971853	MG972002	NA	MG971659	NA
*Cytosporaatrocirrhata*	CFCC 89615	*Juglansregia*	China	KR045618	KF498673	KU710946	KP310858	KR045659
*Cytosporaatrocirrhata*	CFCC 89616	*Juglansregia*	China	KR045619	KF498674	KU710947	KP310859	KR045660
*Cytosporabeilinensis*	CFCC 50493^T^	*Pinusarmandii*	China	MH933619	MH933527	NA	MH933495	MH933561
*Cytosporabeilinensis*	CFCC 50494	*Pinusarmandii*	China	MH933620	MH933528	NA	MH933496	MH933562
*Cytosporaberberidis*	CFCC 89927^T^	*Berberisdasystachya*	China	KR045620	KU710990	KU710948	KU710913	KR045661
*Cytosporaberberidis*	CFCC 89933	*Berberisdasystachya*	China	KR045621	KU710991	KU710949	KU710914	KR045662
*Cytosporabungeana*	CFCC 50495^T^	*Pinusbungeana*	China	MH933621	MH933529	MH933593	MH933497	MH933563
*Cytosporabungeana*	CFCC 50496	*Pinusbungeana*	China	MH933622	MH933530	MH933594	MH933498	MH933564
*Cytosporacalifornica*	CBS 144234^T^	*Juglansregia*	USA	MG971935	MG972083	NA	MG971645	NA
*Cytosporacarbonacea*	CFCC 89947	*Ulmuspumila*	China	KR045622	KP310842	KU710950	KP310855	KP310825
*Cytosporacarpobroti*	CMW48981^T^	*Carpobrotusedulis*	South Africa	MH382812	NA	NA	MH411212	MH411207
*Cytosporaceltidicola*	CFCC 50497^T^	*Celtissinensis*	China	MH933623	MH933531	MH933595	MH933499	MH933566
*Cytosporaceltidicola*	CFCC 50498	*Celtissinensis*	Anhui, China	MH933624	MH933532	MH933596	MH933500	MH933567
*Cytosporacentrivillosa*	MFLUCC 16-1206^T^	*Sorbusdomestica*	Italy	MF190122	NA	MF377600	NA	NA
*Cytosporacentrivillosa*	MFLUCC 17-1660	*Sorbusdomestica*	Italy	MF190123	NA	MF377601	NA	NA
*Cytosporaceratosperma*	CFCC 89624	*Juglansregia*	China	KR045645	NA	KU710976	KP310860	KR045686
*Cytosporaceratosperma*	CFCC 89625	*Juglansregia*	China	KR045646	NA	KU710977	KP310861	KR045687
*Cytosporaceratospermopsis*	CFCC 89626^T^	*Juglansregia*	China	KR045647	KU711011	KU710978	KU710934	KR045688
*Cytosporaceratospermopsis*	CFCC 89627	*Juglansregia*	China	KR045648	KU711012	KU710979	KU710935	KR045689
*Cytosporachrysosperma*	CFCC 89629	*Salixpsammophila*	China	KF765673	NA	KF765705	NA	NA
*Cytosporachrysosperma*	CFCC 89981	*Populusalba*	China	MH933625	MH933533	MH933597	MH933501	MH933568
*Cytosporachrysosperma*	CFCC 89982	*Ulmuspumila*	China	KP281261	KP310835	NA	KP310848	KP310818
*Cytosporacinnamomea*	CFCC 53178^T^	*Prunusarmeniaca*	China	MK673054	MK673024	NA	NA	MK672970
*Cytosporacoryli*	CFCC 53162^T^	*Corylusmandshurica*	China	MN854450	NA	MN850751	MN850758	MN861120
*Cytosporacorylina*	CFCC 54684^T^	*Corylusheterophylla*	China	MW839861	MW815937	MW815951	MW815886	MW883969
*Cytosporacorylina*	CFCC 54685	*Corylusheterophylla*	China	MW839862	MW815938	MW815952	MW815887	MW883970
*Cytosporacotini*	MFLUCC 14-1050^T^	*Cotinuscoggygria*	Russia	KX430142	NA	KX430144	NA	NA
*Cytosporacotoneastricola*	CF 20197027	*Cotoneaster* sp.	China	MK673072	MK673042	MK673012	MK672958	MK672988
*Cytosporacotoneastricola*	CF 20197028	*Cotoneaster* sp.	China	MK673073	MK673043	MK673013	MK672959	MK672989
*Cytosporacurvata*	MFLUCC 15-0865^T^	*Salixalba*	Russia	KY417728	KY417694	NA	NA	NA
*Cytosporacurvispora*	CFCC 54000^T^	*Corylusheterophylla*	China	MW839851	MW815931	MW815945	MW815880	MW883963
*Cytosporacurvispora*	CFCC 54001	*Corylusheterophylla*	China	MW839853	MW815932	MW815946	MW815881	MW883964
*Cytosporadavidiana*	CXY 1350^T^	*Populusdavidiana*	China	KM034870	NA	NA	NA	NA
*Cytosporadiopuiensis*	CFCC 55479	*Euonymusjaponicus*	China	OQ344753	OQ410625	OQ398735	OQ398762	OQ398791
*Cytosporadiopuiensis*	CFCC 55527	*Euonymusjaponicus*	China	OQ344754	OQ410626	OQ398736	OQ398763	OQ398792
*Cytosporadiscotoma*	CFCC 53137 ^T^	*Platycladusorientalis*	China	MW418404	MW422897	MW422907	MW422919	MW422931
*Cytosporadiscotoma*	CFCC 54368	*Platycladusorientalis*	China	MW418405	MW422898	MW422908	MW422920	MW422932
*Cytosporadonetzica*	MFLUCC 15-0864	*Crataegusmonogyna*	Russia	KY417729	KY417695	KY417797	NA	NA
*Cytosporadonetzica*	MFLUCC 16-0574^T^	*Crataegusmonogyna*	Russia	KY417731	KY417697	KY417799	NA	NA
*Cytosporadonglingensis*	CFCC 53159 ^T^	*Platycladusorientalis*	China	MW418412	MW422903	MW422915	MW422927	MW422939
*Cytosporadonglingensis*	CFCC 53160	*Platycladusorientalis*	China	MW418414	MW422905	MW422917	MW422929	MW422941
*Cytosporaelaeagni*	CFCC 89632	*Elaeagnusangustifolia*	China	KR045626	KU710995	KU710955	KU710918	KR045667
*Cytosporaelaeagni*	CFCC 89633	*Elaeagnusangustifolia*	China	KF765677	KU710996	KU710956	KU710919	KR045668
*Cytosporaelaeagnicola*	CFCC 52882^T^	*Elaeagnusangustifolia*	China	MK732341	MK732344	MK732347	NA	NA
*Cytosporaelaeagnicola*	CFCC 52883	*Elaeagnusangustifolia*	China	MK732342	MK732345	MK732348	NA	NA
*Cytosporaerumpens*	CFCC 50022	*Prunuspadus*	China	MH933627	MH933534	NA	MH933502	MH933569
*Cytosporaerumpens*	CFCC 53163	*Prunuspadus*	China	MK673059	MK673029	MK673000	MK672948	MK672975
*Cytosporaeucalypti*	CBS 144241	*Eucalyptusglobulus*	USA	MG971907	MG972056	NA	MG971617	NA
*Cytosporaeuonymicola*	CFCC 50499^T^	*Euonymuskiautschovicus*	China	MH933628	MH933535	MH933598	MH933503	MH933570
*Cytosporaeuonymicola*	CFCC 50500	*Euonymuskiautschovicus*	China	MH933629	MH933536	MH933599	MH933504	MH933571
*Cytosporaeuonymina*	CFCC 89993^T^	*Euonymuskiautschovicus*	China	MH933630	MH933537	MH933600	MH933505	MH933590
*Cytosporaeuonymina*	CFCC 89999	*Euonymuskiautschovicus*	China	MH933631	MH933538	MH933601	MH933506	MH933591
*Cytosporafraxinigena*	MFLU 17-0880 ^T^	*Fraxinusornus*	Italy	NR154859	NA	NA	NA	NA
*Cytosporafugax*	CXY 1371	*Populussimonii*	China	KM034852	NA	NA	NA	KM034891
*Cytosporafugax*	CXY 1381	*Populusussuriensis*	China	KM034853	NA	NA	NA	KM034890
*Cytosporafusispora*	NFCCI 4372	NA	India	MN227694	NA	NA	NA	NA
*Cytosporagalegicola*	MFLUCC 18-1199^T^	*Galegaofficinalis*	Italy	MK912128	MN685810	MN685820	NA	NA
*Cytosporagigalocus*	CFCC 89620^T^	*Juglansregia*	China	KR045628	KU710997	KU710957	KU710920	KR045669
*Cytosporagigalocus*	CFCC 89621	*Juglansregia*	China	KR045629	KU710998	KU710958	KU710921	KR045670
*Cytosporagigaspora*	CFCC 50014	*Juniperusprocumbens*	China	KR045630	KU710999	KU710959	KU710922	KR045671
*Cytosporagigaspora*	CFCC 89634^T^	*Salixpsammophila*	China	KF765671	KU711000	KU710960	KU710923	KR045672
*Cytosporaglobosa*	MFLU 16-2054^T^	*Abiesalba*	Italy	MT177935	NA	MT432212	MT454016	NA
*Cytosporagranati*	CBS 144237^T^	*Punicagranatum*	USA	MG971799	MG971949	NA	MG971514	NA
*Cytosporahaidianensis*	CFCC 54056	*Euonymusalatus*	China	MT360041	MT363978	MT363987	MT363997	MT364007
*Cytosporahaidianensis*	CFCC 54057^T^	*Euonymusalatus*	China	MT360042	MT363979	MT363988	MT363998	MT364008
*Cytosporahippophaës*	CFCC 89639	*Hippophaërhamnoides*	China	KR045632	KU711001	KU710961	KU710924	KR045673
*Cytosporahippophaës*	CFCC 89640	*Hippophaërhamnoides*	China	KF765682	KF765730	KU710962	KP310865	KR045674
*Cytosporajaponica*	CFCC 89956	*Prunuscerasifera*	China	KR045624	KU710993	KU710953	KU710916	KR045665
*Cytosporajaponica*	CFCC 89960	*Prunuscerasifera*	China	KR045625	KU710994	KU710954	KU710917	KR045666
*Cytosporajoaquinensis*	CBS 144235	*Populusdeltoides*	USA	MG971895	MG972044	NA	MG971605	NA
*Cytosporajunipericola*	MFLU 17-0882^T^	*Juniperuscommunis*	Italy	MF190125	NA	NA	MF377580	NA
*Cytosporajuniperina*	CFCC 50501^T^	*Juniperusprzewalskii*	China	MH933632	MH933539	MH933602	MH933507	NA
*Cytosporajuniperina*	CFCC 50502	*Juniperusprzewalskii*	China	MH933633	MH933540	MH933603	MH933508	MH933572
*Cytosporakantschavelii*	CXY 1383	*Populusmaximowiczii*	China	KM034867	NA	NA	NA	NA
*Cytosporakuanchengensis*	CFCC 52464^T^	*Castaneamollissima*	China	MK432616	MK442940	MK578076	NA	NA
*Cytosporakuanchengensis*	CFCC 52465	*Castaneamollissima*	China	MK432617	MK442941	MK578077	NA	NA
*Cytosporaleucosperma*	CFCC 89622	*Pyrusbretschneideri*	China	KR045616	KU710988	KU710944	KU710911	KR045657
*Cytosporaleucosperma*	CFCC 89894	*Pyrusbretschneideri*	China	KR045617	KU710989	KU710945	KU710912	KR045658
*Cytosporalongispora*	CBS 144236^T^	*Prunusdomestica*	USA	MG971905	MG972054	NA	MG971615	NA
*Cytosporalongistiolata*	MFLUCC 16-0628	Salix×fragilis	Russia	KY417734	KY417700	KY417802	NA	NA
*Cytosporalumnitzericola*	MFLUCC 17-0508^T^	*Lumnitzeraracernosa*	Tailand	MG975778	MH253457	MH253461	NA	NA
*Cytosporamali*	CFCC 50028	*Maluspumila*	China	MH933641	MH933548	MH933606	MH933513	MH933577
*Cytosporamali*	CFCC 50029	*Maluspumila*	China	MH933642	MH933549	MH933607	MH933514	MH933578
*Cytosporamali*-*spectabilis*	CFCC 53181^T^	*Malusspectabilis*	China	MK673066	MK673036	MK673006	MK672953	MK672982
*Cytosporamelnikii*	CFCC 89984	*Rhustyphina*	China	MH933644	MH933551	MH933609	MH933515	MH933580
***Cytosporamyricicola***	**CFCC 59323^T^**	***Myricariapaniculate***	**China**	**OR769868**	**OR767324**	**OR767338**	**OR767364**	**OR767351**
***Cytosporamyricicola***	**CFCC 59324**	***Myricariapaniculate***	**China**	**OR769869**	**OR767325**	**OR767339**	**OR767365**	**OR767352**
***Cytosporamyricicola***	**CFCC 59325**	***Myricariapaniculate***	**China**	**OR769870**	**OR767326**	**OR767340**	**OR767366**	**OR767353**
*Cytosporamyrtagena*	CFCC 52454	*Castaneamollissima*	China	MK432614	MK442938	MK578074	NA	NA
*Cytosporamyrtagena*	CFCC 52455	*Castaneamollissima*	China	MK432615	MK442939	MK578075	NA	NA
*Cytosporanivea*	MFLUCC 15-0860	*Salixacutifolia*	Russia	KY417737	KY417703	KY417805	NA	NA
*Cytosporanivea*	CFCC 89641	*Elaeagnusangustifolia*	China	KF765683	KU711006	KU710967	KU710929	KR045679
*Cytosporanotastroma*	NE_TFR5	*Populustremuloides*	USA	JX438632	NA	NA	JX438543	NA
*Cytosporanotastroma*	NE_TFR8	*Populustremuloides*	USA	JX438633	NA	NA	JX438542	NA
*Cytosporaochracea*	CFCC 53164^T^	*Cotoneaster* sp.	China	MK673060	MK673030	MK673001	MK672949	MK672976
*Cytosporaoleicola*	CBS 144248^T^	*Oleaeuropaea*	USA	MG971944	MG972098	NA	MG971660	NA
*Cytosporaolivacea*	CFCC 53174	*Prunuscerasifera*	China	MK673058	MK673028	MK672999	NA	MK672974
*Cytosporaolivacea*	CFCC 53175	*Prunusdulcis*	China	MK673062	MK673032	MK673003	NA	MK672978
*Cytosporapalm*	CXY 1276	*Cotinuscoggygria*	China	JN402990	NA	NA	KJ781296	NA
*Cytosporapalm*	CXY 1280^T^	*Cotinuscoggygria*	China	JN411939	NA	NA	KJ781297	NA
*Cytosporaparacinnamomea*	CFCC 55453^T^	*Salixmatsudana*	China	MZ702594	OK303456	OK303515	OK303576	OK303643
*Cytosporaparacinnamomea*	CFCC 55455^T^	*Salixmatsudana*	China	MZ702598	OK303460	OK303519	OK303580	OK303647
*Cytosporaparakantschavelii*	MFLUCC 15-0857^T^	Populus×sibirica	Russia	KY417738	KY417704	KY417806	NA	NA
*Cytosporaparapistaciae*	CBS 144506^T^	*Pistaciavera*	USA	MG971804	MG971954	NA	MG971519	NA
*Cytosporaparaplurivora*	FDS-439	*Prunusarmeniaca*	Canada	OL640182	OL631586	NA	OL631589	NA
*Cytosporaparaplurivora*	FDS-564^T^	Prunuspersicavar.nucipersica	Canada	OL640183	OL631587	NA	OL631590	NA
*Cytosporaparasitica*	CFCC 53173	*Berberis* sp.	China	MK673070	MK673040	MK673010	MK672957	MK672986
*Cytosporaparatranslucens*	MFLUCC 15-0506^T^	Populusalbavar.bolleana	Russia	KY417741	KY417707	KY417809	NA	NA
*Cytosporaparatranslucens*	MFLUCC 16-0627	*Populusalba*	Russia	KY417742	KY417708	KY417810	NA	NA
*Cytosporaphialidica*	MFLUCC 17-2498	*Alnusglutinosa*	Italy	MT177932	NA	MT432209	MT454014	NA
*Cytosporapiceae*	CFCC 52841^T^	*Piceacrassifolia*	China	MH820398	MH820406	MH820395	MH820402	MH820387
*Cytosporapiceae*	CFCC 52842	*Piceacrassifolia*	China	MH820399	MH820407	MH820396	MH820403	MH820388
*Cytosporapingbianensis*	MFLUCC 18-1204^T^	Undefined wood	China	MK912135	MN685817	MN685826	NA	NA
*Cytosporapistaciae*	CBS 144238^T^	*Pistaciavera*	USA	MG971802	MG971952	NA	MG971517	NA
*Cytosporaplatycladi*	CFCC 50504^T^	*Platycladusorientalis*	China	MH933645	MH933552	MH933610	MH933516	MH933581
*Cytosporaplatycladi*	CFCC 50505	*Platycladusorientalis*	China	MH933646	MH933553	MH933611	MH933517	MH933582
*Cytosporaplatycladicola*	CFCC 50038^T^	*Platycladusorientalis*	China	KT222840	MH933555	MH933613	MH933519	MH933584
*Cytosporaplatycladicola*	CFCC 50039	*Platycladusorientalis*	China	KR045642	KU711008	KU710973	KU710931	KR045683
*Cytosporaplurivora*	CBS 144239^T^	*Oleaeuropaea*	USA	MG971861	MG972010	NA	MG971572	NA
*Cytosporapopuli*	CFCC 55472 ^T^	*Populus* sp.	China	MZ702609	OK303471	OK303530	OK303591	OK303658
*Cytosporapopuli*	CFCC 55473	*Populus* sp.	China	MZ702610	OK303472	OK303531	OK303592	OK303659
*Cytosporapopulicola*	CBS 144240	*Populusdeltoides*	USA	MG971891	MG972040	NA	MG971601	NA
*Cytosporapopulina*	CFCC 89644^T^	*Salixpsammophila*	China	KF765686	KU711007	KU710969	KU710930	KR045681
***Cytosporapopulina***	**CFCC 58856**	***Prunuscerasifera***	**China**	**OR769873**	**OR767329**	**OR767343**	**OR767369**	**NA**
*Cytosporapopulinopsis*	CFCC 50032^T^	*Sorbusaucuparia*	China	MH933648	MH933556	MH933614	MH933520	MH933585
*Cytosporapopulinopsis*	CFCC 50033	*Sorbusaucuparia*	China	MH933649	MH933557	MH933615	MH933521	MH933586
*Cytosporapredappioensis*	MFLUCC 17-2458^T^	*Platanushybrida*	Italy	MG873484	NA	NA	NA	NA
*Cytosporapredappioensis*	MFLU 17-0327	*Platanushybrida*	Italy	MH253451	MH253449	MH253450	NA	NA
*Cytosporaprunicola*	MFLU 17-0995^T^	*Prunus* sp.	Italy	MG742350	MG742353	MG742352	NA	NA
*Cytosporapruni*-*mume*	CFCC 53179	*Prunusarmeniaca*	China	MK673057	MK673027	NA	MK672947	MK672973
*Cytosporapruni*-*mume*	CFCC 53180^T^	*Prunusmume*	China	MK673067	MK673037	MK673007	MK672954	MK672983
*Cytosporapruinopsis*	CFCC 50034^T^	*Ulmuspumila*	China	KP281259	KP310836	KU710970	KP310849	KP310819
*Cytosporapruinopsis*	CFCC 53153	*Ulmuspumila*	China	MN854451	MN850763	MN850752	MN850759	MN861121
*Cytosporapruinosa*	CFCC 50036	*Syringaoblata*	China	KP310800	KP310832	NA	KP310845	KP310815
*Cytosporapruinosa*	CFCC 50037	*Syringaoblata*	China	MH933650	MH933558	NA	MH933522	MH933589
*Cytosporapubescentis*	MFLUCC 18-1201^T^	*Quercuspubescens*	Italy	MK912130	MN685812	MN685821	NA	NA
*Cytosporapunicae*	CBS 144244	*Punicagranatum*	USA	MG971943	MG972091	NA	MG971654	NA
*Cytosporaquercicola*	MFLU 17-0881	*Quercus* sp.	Italy	MF190128	NA	NA	NA	NA
*Cytosporaribis*	CFCC 50026	*Ulmuspumila*	China	KP281267	KP310843	KU710972	KP310856	KP310826
*Cytosporaribis*	CFCC 50027	*Ulmuspumila*	China	KP281268	KP310844	NA	KP310857	KP310827
*Cytosporarosae*	MFLU 17-0885	*Rosacanina*	Italy	MF190131	NA	NA	NA	NA
*Cytosporarosicola*	CF 20197024^T^	*Rosa* sp.	China	MK673079	MK673049	MK673019	MK672965	MK672995
*Cytosporarosigena*	MFLUCC 18-0921^T^	*Rosa* sp.	Russia	MN879872	NA	NA	NA	NA
*Cytosporarostrata*	CFCC 89909	*Salixcupularis*	China	KR045643	KU711009	KU710974	KU710932	KR045684
*Cytosporarostrata*	CFCC 89910	*Salixcupularis*	China	KR045644	KU711010	KU710975	KU710933	NA
*Cytosporarusanovii*	MFLUCC 15-0853	Populus×sibirica	Russia	KY417743	KY417709	KY417811	NA	NA
*Cytosporarusanovii*	MFLUCC 15-0854^T^	*Salixbabylonica*	Russia	KY417744	KY417710	KY417812	NA	NA
*Cytosporasalicacearum*	MFLUCC 15-0509	*Salixalba*	Russia	KY417746	KY417712	KY417814	NA	NA
*Cytosporasalicacearum*	MFLUCC 15-0861	Salix×fragilis	Russia	KY417745	KY417711	KY417813	NA	NA
*Cytosporasalicicola*	MFLUCC 14-1052^T^	*Salixalba*	Russia	KU982636	KU982637	NA	NA	NA
*Cytosporasalicicola*	MFLUCC 15-0866	*Salix* sp.	Thailand	KY417749	KY417715	KY417817	NA	NA
*Cytosporasalicina*	MFLUCC 15-0862	*Salixalba*	Russia	KY417750	KY417716	KY417818	NA	NA
*Cytosporasalicina*	MFLUCC 16-0637	Salix×fragilis	Russia	KY417751	KY417717	KY417819	NA	NA
*Cytosporaschulzeri*	CFCC 50042	*Maluspumila*	China	KR045650	KU711014	KU710981	KU710937	KR045691
*Cytosporasibiraeae*	CFCC 50045^T^	*Sibiraeaangustata*	China	KR045651	KU711015	KU710982	KU710938	KR045692
*Cytosporasibiraeae*	CFCC 50046	*Sibiraeaangustata*	China	KR045652	KU711016	KU710983	KU710939	KR045693
***Cytosporasibiraeicola***	**CFCC 59100^T^**	***Sibiraeaangustata***	**China**	**OR769871**	**OR767327**	**OR767341**	**OR767367**	**OR767354**
***Cytosporasibiraeicola***	**CFCC 59101**	***Sibiraeaangustata***	**China**	**OR769872**	**OR767328**	**OR767342**	**OR767368**	**OR767355**
*Cytosporasophorae*	CFCC 50047	*Styphnolobiumjaponicum*	China	KR045653	KU711017	KU710984	KU710940	KR045694
*Cytosporasophorae*	CFCC 89598	*Styphnolobiumjaponicum*	China	KR045654	KU711018	KU710985	KU710941	KR045695
*Cytosporasophoricola*	CFCC 89596	*Styphnolobiumjaponicum*	China	KR045656	KU711020	KU710987	KU710943	KR045697
*Cytosporasophoricola*	CFCC 89595^T^	*Styphnolobiumjaponicum*	China	KR045655	KU711019	KU710986	KU710942	KR045696
*Cytosporasophoriopsis*	CFCC 55469	*Salixmatsudana*	China	MZ702583	OK303445	OK303504	OK303565	OK303632
*Cytosporasophoriopsis*	CFCC 89600	*Styphnolobiumjaponicum*	China	KR045623	KU710992	KU710951	KU710915	KP310817
*Cytosporasorbi*	MFLUCC 16-0631^T^	*Sorbusaucuparia*	Russia	KY417752	KY417718	KY417820	NA	NA
*Cytosporasorbicola*	MFLUCC 16-0584^T^	*Acerpseudoplatanus*	Russia	KY417755	KY417721	KY417823	NA	NA
*Cytosporasorbicola*	MFLUCC 16-0633	*Cotoneastermelanocarpus*	Russia	KY417758	KY417724	KY417826	NA	NA
*Cytosporasorbina*	CF 20197660^T^	*Sorbustianschanica*	China	MK673052	MK673022	NA	MK672943	MK672968
*Cytosporaspiraeae*	CFCC 50049^T^	*Spiraeasalicifolia*	China	MG707859	MG708196	MG708199	NA	NA
*Cytosporaspiraeae*	CFCC 50050	*Spiraeasalicifolia*	China	MG707860	MG708197	MG708200	NA	NA
*Cytosporaspiraeicola*	CFCC 53138^T^	*Spiraeasalicifolia*	China	MN854448	NA	MN850749	MN850756	MN861118
*Cytosporaspiraeicola*	CFCC 53139	*Tilianobilis*	China	MN854449	NA	MN850750	MN850757	MN861119
*Cytosporatamaricicola*	CFCC 50507	*Rosamultifolora*	China	MH933651	MH933559	MH933616	MH933525	MH933587
*Cytosporatamaricicola*	CFCC 50508^T^	*Tamarixchinensis*	China	MH933652	MH933560	MH933617	MH933523	MH933588
*Cytosporatanaitica*	MFLUCC 14-1057^T^	*Betulapubescens*	Russia	KT459411	KT459413	NA	NA	NA
*Cytosporathailandica*	MFLUCC 17-0262^T^	*Xylocarpusmoluccensis*	Thailand	MG975776	MH253459	MH253463	NA	NA
*Cytosporathailandica*	MFLUCC 17-0263^T^	*Xylocarpusmoluccensis*	Thailand	MG975777	MH253460	MH253464	NA	NA
*Cytosporatibetensis*	CF 20197026	*Cotoneaster* sp.	China	MK673076	MK673046	MK673016	MK672962	MK672992
*Cytosporatibetensis*	CF 20197029	*Cotoneaster* sp.	China	MK673077	MK673047	MK673017	MK672963	MK672993
*Cytosporatibouchinae*	CPC 26333^T^	*Tibouchinasemidecandra*	France	KX228284	NA	NA	NA	NA
*Cytosporatranslucens*	CXY 1351	*Populusdavidiana*	China	KM034874	NA	NA	NA	KM034895
*Cytosporatranslucens*	CXY 1359	*Populus* × beijingensis	China	KM034871	NA	NA	NA	KM034894
*Cytosporaulmi*	MFLUCC 15-0863^T^	*Ulmusminor*	Russia	KY417759	NA	NA	NA	NA
*Cytosporaverrucosa*	CFCC 53157 ^T^	*Platycladusorientalis*	China	MW418408	NA	MW422911	MW422923	MW422935
*Cytosporaverrucosa*	CFCC 53158	*Platycladusorientalis*	China	MW418410	MW422901	MW422913	MW422925	MW422937
*Cytosporavinacea*	CBS 141585^T^	*Vitisinterspecific*	USA	KX256256	NA	NA	KX256277	KX256235
*Cytosporaviridistroma*	CBS 202.36^T^	*Cerciscanadensis*	USA	MN172408	NA	NA	MN271853	NA
*Cytosporaviticola*	Cyt2	*Vitisinterspecific*	USA	KX256238	NA	NA	KX256259	KX256217
*Cytosporaviticola*	CBS 141586^T^	*Vitisvinifera*	USA	KX256239	NA	NA	KX256260	KX256218
*Cytosporaxinjiangensis*	CFCC 53182	*Rosa* sp.	China	MK673064	MK673034	MK673004	MK672951	MK672980
*Cytosporaxinjiangensis*	CFCC 53183^T^	*Rosa* sp.	China	MK673065	MK673035	MK673005	MK672952	MK672981
*Cytosporaxinglongensis*	CFCC 52458	*Castaneamollissima*	China	MK432622	MK442946	MK578082	NA	NA
*Cytosporaxinglongensis*	CFCC 52459	*Castaneamollissima*	China	MK432623	MK442947	MK578083	NA	NA
*Cytosporaxylocarpi*	MFLUCC 17-0251^T^	*Xylocarpusgranatum*	Thailand	MG975775	MH253458	MH253462	NA	NA
*Cytosporazhaitangensis*	CFCC 56227 ^T^	*Euonymusjaponicus*	China	OQ344750	OQ410623	OQ398733	OQ398760	OQ398789
*Cytosporazhaitangensis*	CFCC 57537	*Euonymusjaponicus*	China	OQ344751	OQ410624	OQ398734	OQ398761	OQ398790
*Diaporthevaccinii*	CBS 160.32	*Vacciniummacrocarpon*	USA	KC343228	JQ807297	NA	KC343954	KC344196

Ex-type strains are indicated with (^T^) after the collection number; “NA” indicates unavailable sequences; sequences produced in the current study are in bold.

### ﻿Taxonomy

#### ﻿ Cytospora myricicola

Taxon classificationFungiDiaporthalesCytosporaceae

Ning Jiang sp. nov.

01B68910-8BBE-5176-B1E0-8BC5255B820F

MycoBank No: 850240

Fig. 2

##### Etymology.

“*myrici*” refers to the host genus *Myricaria* and “-*cola*” means inhabiting.

**Figure 2.**
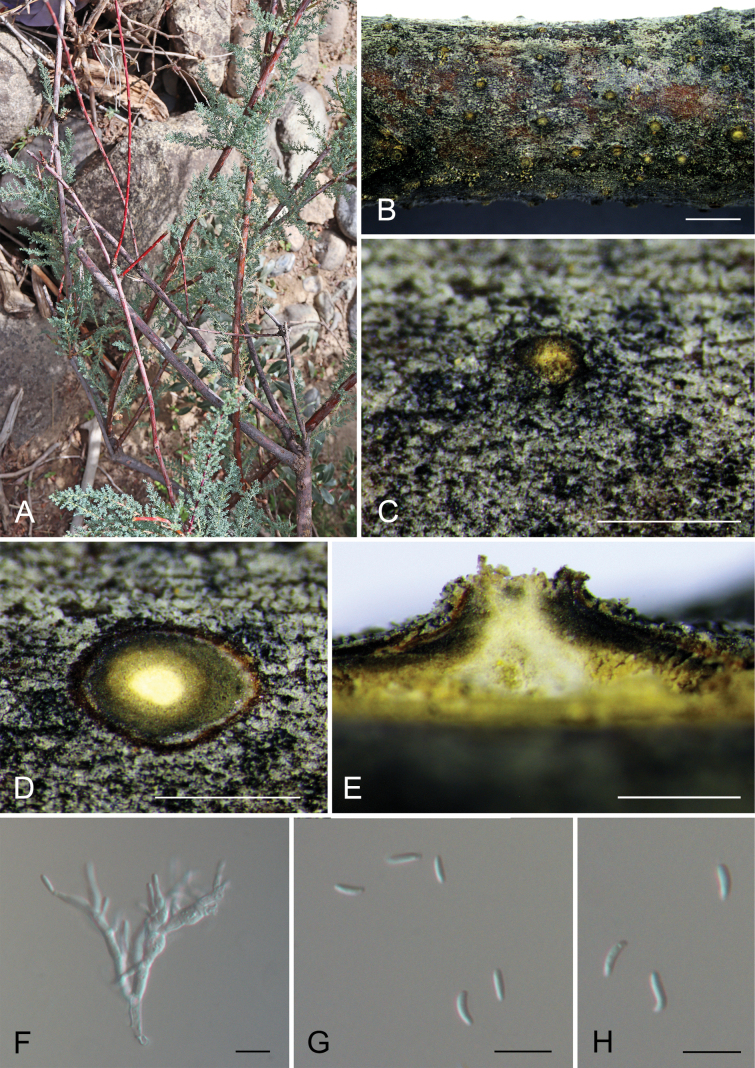
*Cytosporamyricicola* (CAF800083, holotype) **A, B** canker disease symptom **C** conidioma **D** transverse section through a conidioma **E** longitudinal section through a conidioma **F** conidiophores and conidia **G, H** conidia. Scale bars: 2000 µm (**B**); 1000 µm (**C, D**); 500 µm (**E**); 10 µm (**F–H**).

##### Holotype.

CAF800083.

##### Description.

Associated with branch canker disease of *Myricariapaniculate*. ***Sexual morph***: Undetermined. ***Asexual morph***: Pycnidial stromata ostiolated, semi-immersed in the host bark, scattered, discoid, with multiple locules. Conceptacle dark brown to black, circular surrounded stromata. Ectostromatic disc dark yellow, circular to ovoid, (250–)350–450(–550) μm diam., with one ostiole per disc. Ostioles dark, at the same level as the disc, (35–)55–85(–100) μm diam. Locule numerous, arranged circularly or elliptically with independent walls (245–)300–450(–550) μm diam. Peridium comprising a few layers of cells of textura angularis, with innermost layer brown, outer layer brown to dark brown. Conidiophores hyaline, branched, thin-walled, filamentous. Conidiogenous cells enteroblastic polyphialidic, 6.5–35.5 × 1.5–2.5 μm. Conidia hyaline, allantoid, smooth, aseptate, thin-walled, (4.4–)4.7–5.6(–5.8) × 1.4–1.7 μm (x̄ = 5.2 × 1.6 μm).

##### Culture characteristics.

Colonies on PDA flat, with flocculent aerial mycelium and entire edge, initially white, becoming dark and reaching 90 mm diameter after 10 days at 25 °C, sterile.

##### Materials examined.

China, Tibet Tibetan Autonomous Region, Lhasa City, Mozhugongka County, Riduo Township, Zen Village, on cankered branches of *Myricariapaniculate*, 28 July 2022, Jin Peng, Liu Yuanyuan, Jiang Ning and Liu Min (CAF800083, ***holotype***); ex-type culture CFCC 59323. China, Tibet Tibetan Autonomous Region, Lhasa City, Mozhugongka County, Riduo Township, Renqinglin Village, on cankered branches of *Myricariapaniculate*, 28 July 2022, Jin Peng, Liu Yuanyuan, Jiang Ning and Liu Min (XZ010B); cultures CFCC 59324 and CFCC 59325.

##### Notes.

Phylogenetically, *Cytosporamyricicola* is close to *C.fraxinigena*, *C.junipericola*, *C.pubescentis*, *C.quercicola* and *C.rosae* (Fig. 1). Of these six species, only *C.myricicola*, *C.pubescentis* and *C.rosae* have asexual morph descriptions; *C.myricicola* (4.7–5.6 × 1.4–1.7 μm) is different from *C.pubescentis* (5.8–7.5 × 1.3–1.6 μm) by shorter conidia and from *C.rosae* (3–5 × 0.5–1 μm) by larger conidia (Senanayake et al. 2017; Shang et al. 2020). In addition, *C.myricicola* can be distinguished from the other five species by host and distribution (*C.myricicola* from *Myricariapaniculate* in China vs. *C.fraxinigena* from *Fraxinusornus* in Italy vs. *C.junipericola* from *Juniperuscommunis* in Italy vs. *C.pubescentis* from *Quercuspubescens* in Italy vs. *C.quercicola* from *Quercus* sp. in Italy vs. *C.rosae* from *Rosacanina* in Italy) (Senanayake et al. 2017; Shang et al. 2020).

#### ﻿ Cytospora populina

Taxon classificationFungiDiaporthalesCytosporaceae

(Pers.) Rabenh., Deutschl. Krypt.-Fl. (Leipzig) 1: 148. 1844

1555AFEA-3347-5D3B-B54A-73345BF03C5D

Fig. 3

##### Description.

Associated with branch canker disease of *Prunuscerasifera*. ***Sexual morph***: Stromata immersed in bark. Ascostromata, erumpent through the surface of bark, lenticular, extending to a large circular area, (750–)900–1200(–1350) μm diam. Disc grey to black, circular to ovoid, (85–)100–150(–195) μm in diameter. Ostioles numerous, dark brown to black, at the same level as the disc, (25–)31–46(–52) μm diam. Locules dark brown, arranged circularly, flask-shaped to spherical, (180–)195–285(–340) μm diam. Asci clavate to elongate obovoid, (45–)55.5–62.5(–67) × (6.5–)8–12(–16) μm, 4-spored. Ascospores biseriate, elongate-allantoid, thin-walled, hyaline, aseptate, (15–)18.5–23.5(–25.5) × (4–)4.5–5.5(–6.5) μm (x̄ = 20.4 × 5.1 μm). ***Asexual morph***: Undetermined.

**Figure 3.**
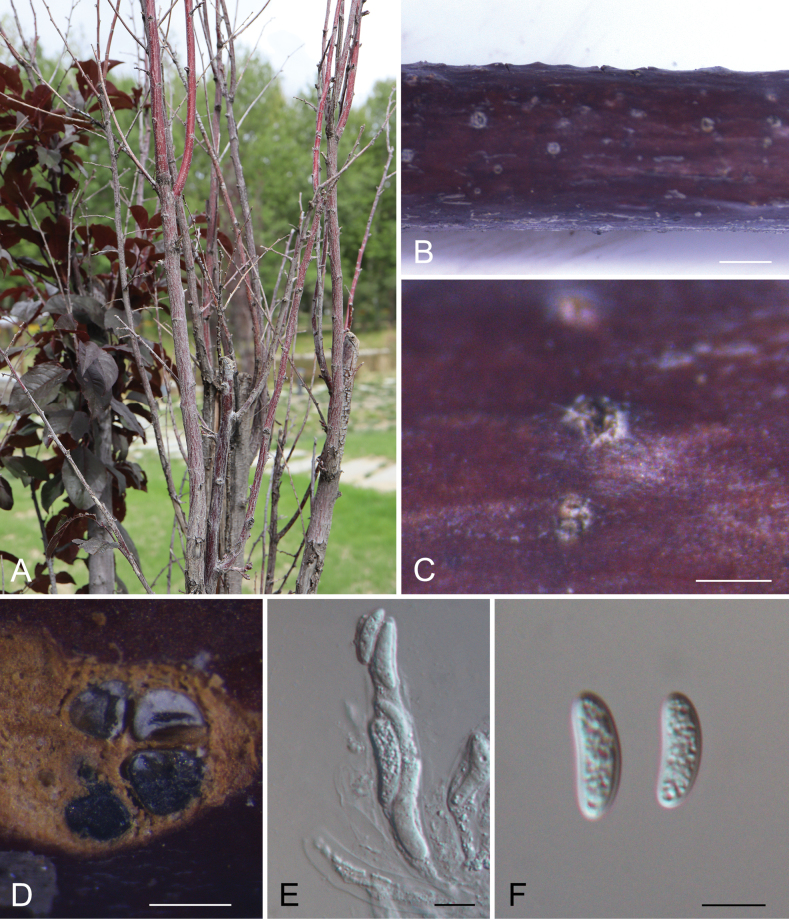
*Cytosporapopulina* (CAF800085) **A, B** canker disease symptom **C** ascostromata **D** transverse section through an ascostroma **E** asci and Ascospores **F** ascospores. Scale bars: 2000 µm (**B**); 500 µm (**C**); 200 µm (**D**); = 10 µm (**E–F**).

##### Culture characteristics.

Colonies on PDA flat, with flocculent aerial mycelium and entire edge, initially white, becoming luteous and reaching 80 mm diameter after 10 days at 25 °C, sterile.

##### Materials examined.

China, Tibet Tibetan Autonomous Region, Shigatse City, Sangzhuzi District, Gongjuelinka Park, on cankered branches of *Prunuscerasifera*, 2 August 2022, Jin Peng, Jiang Ning and Liu Min (XZ063); culture CFCC 58856.

##### Notes.

*Cytosporapopulina* has been reported from *Populuscanadensis* in Argentina, *Salixpsammophila* in Shaanxi Province of China and *Acerpubescens Rubus* sp. in Uzbekistan (Farr 1973; Fan et al. 2015; Gafforov 2017). This fungus is distinguished from the other *Cytospora* species by its 4-ascospored asci and undiscovered asexual state (Fan et al. 2015). In the present study, we firstly found this fungus causing cankered branches of *Prunuscerasifera* in Tibet, China.

#### ﻿ Cytospora sibiraeicola

Taxon classificationFungiDiaporthalesCytosporaceae

Ning Jiang sp. nov.

50058A64-9316-5086-A370-BA91E01FA307

MycoBank No: 850241

Fig. 4

##### Etymology.

“*sibiraei*” refers to the host genus *Sibiraea* and “-*cola*” means inhabiting.

##### Holotype.

CAF800084.

##### Description.

Associated with branch canker disease of *Sibiraeaangustata*. ***Sexual morph***: Undetermined. ***Asexual morph***: Pycnidial stromata ostiolated, immersed or semi-immersed in the host bark, scattered, discoid, with multiple locules. Conceptacle black, circular surrounded stromata. Ectostromatic disc black, circular to ovoid, (200–)300–450(–500) μm diam., with one ostiole per disc. Ostioles dark, at the same level as the disc, (30–)60–80(–95) μm diam. Locule numerous, arranged circularly or elliptically with independent walls, (200–)250–380(–500) μm diam. Peridium comprising few layers of cells of textura angularis, with innermost layer brown, outer layer brown to dark brown. Conidiophores hyaline, unbranched, thin-walled, filamentous. Conidiogenous cells enteroblastic polyphialidic, 12.5–32.5 × 2–3.5 μm. Conidia hyaline, allantoid, smooth, aseptate, thin-walled, (3.3–)3.4–4.3(–4.5) × 1.2–1.6 μm (x̄ = 3.9 × 1.5 μm).

**Figure 4.**
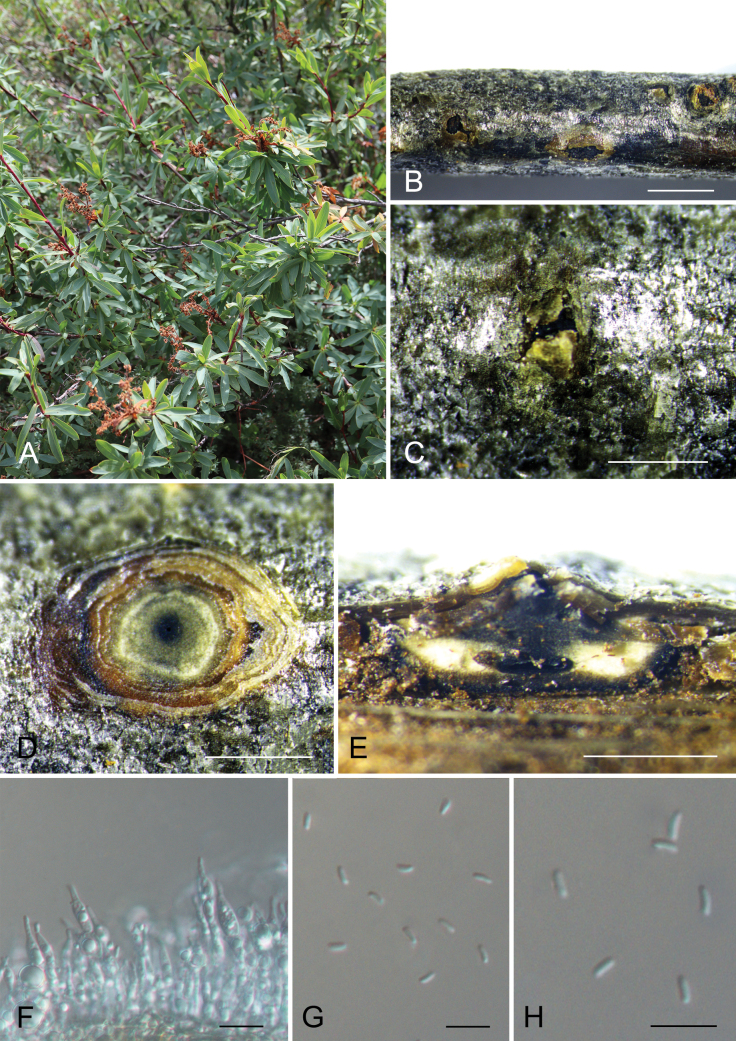
*Cytosporasibiraeicola* (CAF800084, holotype) **A, B** canker disease symptom **C** conidioma **D** transverse section through a conidioma **E** longitudinal section through a conidioma **F** conidiophores and conidia **G, H** conidia. Scale bars: 2000 µm (**B**); 1000 µm (**C–E**); 10 µm (**F–H**).

##### Culture characteristics.

Colonies on PDA flat, with flocculent aerial mycelium and undulate margin, initially white, becoming olivaceous grey and reaching 90 mm diameter after 10 days at 25 °C, sterile.

##### Materials examined.

China, Tibet Tibetan Autonomous Region, Lhasa City, Mozhugongka County, Riduo Township, Zen Village, on cankered branches of *Sibiraeaangustata*, 28 July 2022, Jin Peng, Liu Yuanyuan, Jiang Ning and Liu Min (CAF800083, holotype); ex-type cultures CFCC 59100 and CFCC 59101.

##### Notes.

*Cytosporasibiraeicola* is phylogenetically close to *C.phialidica* and *C.viticola* (Fig. 1). Morphologically, *C.sibiraeicola* (3.4–4.3 × 1.2–1.6 μm) and *C.phialidica* (3.5–5 × 1–2 μm) have much shorter conidia than *C.viticola* (5.2–7 × 0.9–1.6 μm) (Lawrence et al. 2017; Li et al. 2020). In addition, these three species can be distinguished by the host and distribution (*C.sibiraeicola* from *Sibiraeaangustata* in China vs. *C.phialidica* from *Alnusglutinosa* in Italy vs. *C.viticola* from *Vitisvinifera* in the USA) (Lawrence et al. 2017; Li et al. 2020).

## ﻿Discussion

*Cytospora* is a species-rich genus occurring on various plant hosts (Fotouhifar et al. 2010; Aiello et al. 2019; Jayawardena et al. 2019; Úrbez-Torres et al. 2020; Hanifeh et al. 2022). However, in the third pole of the Earth named Qinghai-Tibet Plateau, canker pathogens, such as *Cytospora*, have been seldom surveyed previously. In the comprehensive study on the genus *Cytospora* in China, only one species *C.chrysosperma* was recorded from Ulmuspumila in Tibet (Fan et al. 2020). Subsequently, *Cytosporacotoneastricola* and *C.tibetensis* from *Cotoneaster* sp. and *Cytosporarosicola* from *Rosa* sp. were discovered in Tibet (Pan et al. 2020). The current study introduces two new species named *C.myricicola* from *Myricariapaniculate* and *C.sibiraeicola* from *Sibiraeaangustata* in Tibet, China. In addition, a new host record of *C.populina* on *Prunuscerasifera* was discovered.

To our knowledge, *Cytosporamyricicola* is the first species of *Cytospora* discoved on the host genus *Myricaria* (Fan et al. 2020). *Cytosporasibiraeicola* and *C.sibiraeae* have been recorded from the host species *Sibiraeaangustata* (Liu et al. 2015). *Cytosporasibiraeae* was described, based only on the sexual morph and is currently impossible to be distinguished from *C.sibiraeicola* morphologically (Liu et al. 2015). However, these two species occurring on *Sibiraeaangustata* are phylogenetically obviously distinct (Fig. 1).

Species of *Cytospora* are known as opportunistic pathogens mainly infecting woody hosts and some of the species occur on a wide host range (Adams et al. 2005; Fan et al. 2020). The *Cytospora* species and their host association have been revealed in this study; however, further studies are required to confirm the fungal pathogenicity.

## Supplementary Material

XML Treatment for Cytospora myricicola

XML Treatment for Cytospora populina

XML Treatment for Cytospora sibiraeicola

## ﻿Additional information

### Author ORCIDs

Jiangrong Li https://orcid.org/0000-0002-6679-5227

Jieting Li https://orcid.org/0009-0001-8984-7261

Ning Jiang https://orcid.org/0000-0002-9656-8500

### Data availability

All of the data that support the findings of this study are available in the main text.
